# No Adjuvant Effect of *Bacillus thuringiensis*-Maize on Allergic Responses in Mice

**DOI:** 10.1371/journal.pone.0103979

**Published:** 2014-08-01

**Authors:** Daniela Reiner, Rui-Yun Lee, Gerhard Dekan, Michelle M. Epstein

**Affiliations:** 1 Department of Dermatology, Division of Immunology, Allergy and Infectious Diseases, Medical University of Vienna, Vienna, Austria; 2 Institute of Clinical Pathology, Medical University of Vienna, Vienna, Austria; TGen, United States of America

## Abstract

Genetically modified (GM) foods are evaluated carefully for their ability to induce allergic disease. However, few studies have tested the capacity of a GM food to act as an adjuvant, i.e. influencing allergic responses to other unrelated allergens at acute onset and in individuals with pre-existing allergy. We sought to evaluate the effect of short-term feeding of GM *Bacillus thuringiensis* (*Bt*)-maize (MON810) on the initiation and relapse of allergic asthma in mice. BALB/c mice were provided a diet containing 33% GM or non-GM maize for up to 34 days either before ovalbumin (OVA)-induced experimental allergic asthma or disease relapse in mice with pre-existing allergy. We observed that GM-maize feeding did not affect OVA-induced eosinophilic airway and lung inflammation, mucus hypersecretion or OVA-specific antibody production at initiation or relapse of allergic asthma. There was no adjuvant effect upon GM-maize consumption on the onset or severity of allergic responses in a mouse model of allergic asthma.

## Introduction

The cultivation of genetically modified (GM) plants has increased dramatically worldwide and is correlated with a concomitant rise in GM containing foods for animal and human consumption. In 2012, there were over 170 million biotech crop hectares with the US leading with 69.5 million hectares [1]. One of the most important and broadly grown GM plants is MON810 maize, which expresses *Cry1Ab* protein from *Bacillus thuringiensis* (*Bt*) and confers resistance to the European maize borer [2]–[4]. Last year *Bt*-maize was grown in 17 countries and constituted 35% of global planting with almost 130,000 hectares grown in Europe [1].

Most studies have shown that GM food containing *Bt* are safe for human and animal consumption [5]–[11]. However, there are a few reports demonstrating adverse effects under certain conditions in livestock and animal models [12], [13]. Due to a significant increase in IgE-mediated allergic diseases in the past few decades, the allergenic potential of novel foods including GM food is a public health concern. However, there is no evidence that Cry proteins are allergenic. *Cry1Ab* has no homology to allergenic proteins [14] and when tested in maize-sensitive individuals, extracts of MON810 or pure *Cry1Ab* did not cause reactions in skin prick tests or induce IgE [15]. Furthermore, in related GM food experiments, rats fed *Bt*-rice for 28 or 90 days had no adverse immune effects [16].

There is some suggestion that while consumption of *Bt*-maize may not cause significant direct adverse allergic effects, a few studies suggest that it may augment allergic responses to unrelated allergens. For example, *Cry1Ac* has an apparent adjuvant effect [17]–[19] and *Cry1Ab* was shown to act as an adjuvant by generating increasing Th2 and Th17-cytokine production in airways in an experimental mouse model [20]. Thus, there continues to be uncertainty about the potential of GM food-induced adjuvant effects. However, few studies have tested *in vivo* adjuvanticity of GM foods [21], [22]. We hypothesize that consumption of food containing GM ingredients leads to adjuvant effects. Here, we aimed to assess the adjuvant effect of *Bt*-maize consumption on the initiation and relapse of ovalbumin (OVA)-induced allergic airways disease in experimental mice.

## Materials and Methods

### Ethics Statement

This study was carried out in strict accordance with the guidelines for the Care and Use of Laboratory Animals of the Austrian Ministry of Science. The protocol was approved by the Committee on the Ethics of the Austrian Ministry of Science (Number: GZ: 68.205/0237-II/3b/2010). All painful procedures were performed under anesthesia and all efforts were made to minimize suffering.

### Mice

Four to six week old female BALB/c mice purchased from Charles River Germany maintained in the Veterinary Medicine University of Vienna animal facility on a 12-h light/dark cycle and provided food and water *ad libitum* were used in all experiments. The mice were fed a diet formulation free of chicken egg proteins (SSNIFF Spezialdiäten GmbH, Soest, Germany, see more details below in the section on feeding protocols). Sentinel health reports revealed no evidence of pathogenic organisms.

### Chemicals

For the induction of allergic disease and ELISAs, we used ovalbumin (OVA, grade V, Sigma Chemical Co., St Louis, MO). Anesthesia used was a mixture of Rompun (Bayer AG, Leverkusen, Germany) and Ketanest S (Pfizer GmbH, Vienna, Austria). Cytological evaluation was done using Kwik-Diff (Thermo Fisher Scientific Inc., Pittsburgh, PA, USA). For ELISA assays, we used bovine serum albumin (BSA, Sigma) and fat-free powdered cow’s milk (Maresi, Vienna, Austria), biotinylated Anti-IgG1 and anti-IgG2a detection mAb and streptavidin horseradish peroxidase (Southern biotechnology associates Inc., Birmingham, AL), 3.3′, 5.5′-tetrametylbenzidine (TMB; BD OptEIA™) substrate and biotinyated anti-IgE, IgG1, IgG2a detection mAbs (Becton Dickinson Biosciences, Franklin Lakes, NJ).

### Induction of allergic asthma

Mice were treated to generate both disease initiation and relapse as previously described [23]. Briefly, mice were immunized intraperitoneally (i.p.) with 10 µg of OVA dissolved in 200 µl phosphate buffered saline (PBS) on days 0 and 21. To induce acute disease, we nebulized 1% OVA in PBS (100 mls) by an ultrasonic nebulizer (Aerodyne, Kendall, Neustadt, Germany) for 60 min twice daily on days 28 and 29. To induce a disease relapse, mice induced with acute disease were allowed to recover for at least 3 months until they were re-challenged with aerosolized 1% OVA for 60 min twice daily on days 91 and 92. Naïve mice were age-matched controls that were not immunized.

### Feeding protocols

The nGM isogenic parent line of maize (Pioneer PR34N43) and GM *Bt*-maize (Pioneer PR34N44; event MON810) were grown simultaneously in Valtierra, Navarra, Spain and purchased directly from the farmer. The GM and non-GM (nGM) maize were tested for the presence of the *Cry1Ab* gene by MON810 event-specific PCR, analyzed for aflatoxin (B1, B2, G1, G2), ochratoxin, zearalenone, vomitoxin, T2 toxin, and fumonsin by ELISA, and pesticide residues by testing against a panel of 355 different active substances [24]. Table 1 illustrates the ingredient composition and chemical content of the 33% maize containing diets (with incorporation of either GM-maize or nGM-maize meal). The basal diet used was V1126-000 from SSNIFF provided *ad libitum* (see http://www.ssniff.de/documents/03_katalog_dt_maus_ratte.pdf). All diets had metabolizable energy of 13.9 MJ/kg assessed using a pig formula (http://www.ssniff.com/documents/02_catalogue_general_abbr._engl.pdf). *Bt*-maize contains 0.0013% *Cry1Ab* content [25]. The experimental diets were pelleted by addition of steam resulting in a temperature of the conditioned meal and pellets of about 70°C.

**Table 1:** Ingredient composition and chemical content of experimental diets.

Item	GM diet (%)	nGM diet (%)	*Basal diet (%)
Corn	33.00	33.00	–
Wheat meal	18.00	18.00	–
Potato protein	17.00	17.00	–
Casein (low sodium)	5.00	5.00	–
Brewer′s yeast, dried	1.00	1.00	–
Whey powder	5.00	5.00	–
Soybean	0.00	0.00	–
Sucrose	3.50	3.50	–
Cellulose powder	6.00	6.00	–
Calcium carbonate	0.60	0.60	–
Calcium phosphate - monobasic	1.00	1.00	–
Salt (NaCl)	0.20	0.20	–
Magnesium oxide (MgO)	0.10	0.10	–
Choline chloride	0.30	0.30	–
Calcium propionate	1.00	1.00	–
DL-Methionine	0.20	0.20	–
L-Tryptophan	0.10	0.10	–
Vitamin pre-mixture	1.00	1.00	–
Mineral & trace element pre-mixture	3.00	3.00	–
Sunflower oil	4.00	4.00	–
Chemical composition			
Dry Matter	88.0	88.0	90.4
Crude Protein	22.7	22.7	22.1
Crude Lipids	5.7	5.7	4.5
Crude Fiber	4.4	4.4	4.4
Crude Ash	5.7	5.7	5.7
Calcium	1.10	1.10	1.10
Phosphorous	0.77	0.77	0.77
Sodium	0.22	0.22	0.22
Lysine	1.60	1.60	1.60
Methionine & Cysteine	1.16	1.16	1.16
Threonine	1.15	1.15	1.15
Tryptophan	0.40	0.40	0.40

*The basal diet ingredient composition is unavailable for commercial reasons. However, in descending order the ingredients are: ground wheat, soybean meal & soy protein concentrate, ground corn & middlings, oat middlings, corn gluten meal/feed, dried sugar beet pulp, mineral compounds, soybean oil, amino acids [DL-Met, L-Lys HCl], vitamins & trace elements.

**Figure 1:**
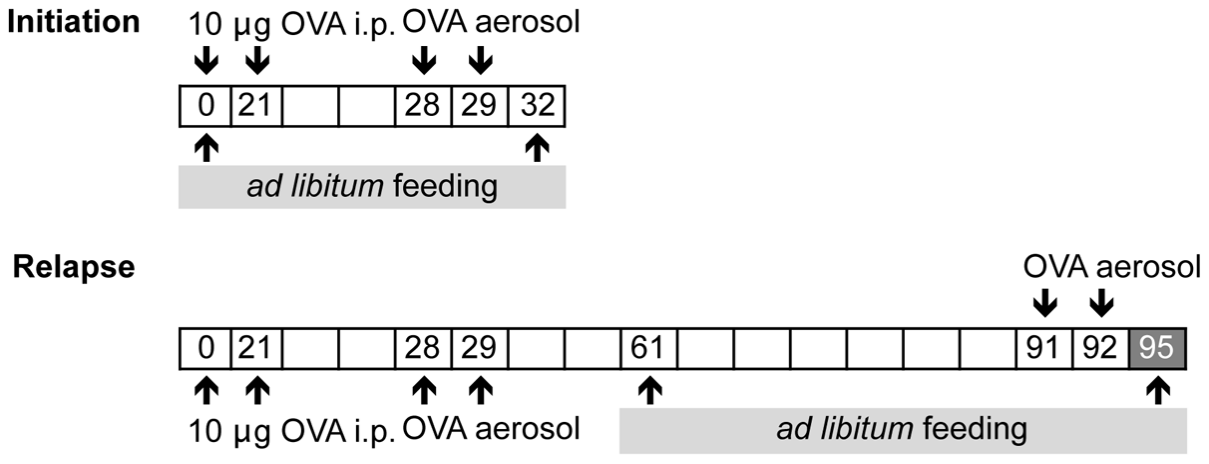
Experimental protocols. *Adjuvant effect of GM-maize on the initiation of OVA-induced allergic lung disease.* Groups of mice were fed either GM-maize, nGM-maize, or basal diets between days 0 and 32 of the initiation of allergic asthma. Mice were immunized to induce allergic disease with 10 µg of OVA on days 0 and 21. One week later, the mice were nebulized with OVA on days 28 and 29. On day 32, antibody titres and allergic lung responses were measured. *Adjuvant effect of GM-maize on OVA-induced allergic disease relapse.* Mice were induced with allergic disease on days 0 and 21 and aerosolized on days 28 and 29 and then allowed to recover for another month (all signs of acute lung and airway inflammation and mucus hypersecretion resolves). Between day 61 and 95, mice were then fed either GM-maize, nGM-maize or basal diets. On days 91 and 92, mice were rechallenged with aerosolized OVA to induce a disease relapse. On day 95, they were evaluated for antibody titres and allergic lung responses.

Mice were fed with diets that were the conventional, basal diet, or diets containing 33% GM or nGM maize prepared by SSNIFF *ad libitum* for 32 and 34 days, respectively. The feeding protocols are illustrated in Figure 1. Feeding of the diets was as follows: To determine whether consumption of a GM diet would influence the initiation of allergic asthma, diets were provided for 32 days from day 0 to 32 during the OVA-sensitization period. To determine whether a GM diet influences a pre-existing allergic disease, mice were fed from day 61 to 95, 34 days after the last aerosol challenge on day 29 and before and during the induction of an OVA-induced disease relapse. The basal diet in this study serves as a secondary control and is meant to represent a conventional mouse diet without the inclusion of maize. The primary control is the nGM maize diet, which is identical in composition to the GM maize diet. Naïve mice were fed a basal diet.

### Airway inflammation

Seventy-two hours after last challenge, mice were terminally anesthetized with a lethal dose of anesthesia, tracheostomy was performed, and a plastic catheter was clamped in the trachea. The lungs were washed 3 times with a total volume of 1 ml PBS and bronchoalveolar lavage fluid (BAL) was collected. The total number of cells in BAL was enumerated in a Neubauer hemocytometer. The percentage of inflammatory cells was determined by morphological examination of at least 300 cells in cytocentrifuged preparations (Cytospin-4, Shandon Instruments, UK) stained with the Kwik-Diff.

### Lung inflammation and mucus secretion

After BAL, lungs were removed, and fixed in 4% paraformaldehyde. Paraffin embedded lung sections were cut and stained with Hematoxylin and Eosin (H&E) for the detection of inflammation, Luna for the specific detection and enumeration of eosinophils, or Periodic-Acid-Schiff (PAS) for analysis of mucus secretion in the goblet cells of the lung epithelium.

A semi-quantitative scoring system was used to grade the severity and extent of inflammation on H&E sections: Severity score was graded as follows: 0 = no inflammation, 1 = inflammation present near central airways, 2 = moderate inflammation, 3 = severe inflammation. Extent of inflammation in the whole lung section was graded as follows: 0 = no inflammation, 1 = <1/3 of inflamed airways, 2 = 1/3–2/3 of inflamed airways, and 3 = >2/3 of inflamed airways. To determine the overall severity of lung inflammation, we multiplied severity and extent of inflammation scores resulting in a range from 0 to 9.

For evaluation of mucus secretion, we used a semi-quantitative scoring system on PAS-stained sections. We graded the presence of mucus within the central and peripheral airway epithelium goblet cell mucus content using as follows: 0 = no mucus detected, 1 = 0–20%, 2 = 21–40%, 3 = 41–60%, 4 = 61–80% and 5 = 81–100% of cells in the airway epithelium were positive for mucus.

### Serum Ag-specific IgG1, IgE and IgG2a

We tested sera taken at the time of evaluation of allergic disease for the measurement of OVA-specific antibodies using standard indirect ELISAs. To measure the titres of OVA-specific Ig, ELISA plates were coated with a solution of OVA dissolved in PBS at 10 µg/ml overnight at 4°C. The plates were washed and blocked with 2% BSA in PBS with 0.05% Tween 20 for 2 h at RT. For BSA and milk IgG1 detection, ELISA plates were coated with a solution of BSA and milk dissolved in PBS at 10 µg/ml overnight at 4°C. The plates were washed and blocked with 50 mM Tris with 0.2% Tween 20 for 2 h at RT. Following coating and blocking, titrated sera were incubated for 24 h at 4°C. After washing, plates were incubated for 2 h at 4°C with either biotinylated anti-IgG1, anti-IgG2a or anti-IgE detection mAbs for OVA and biotinylated anti-IgG1 for BSA and milk, followed by incubation with streptavidin horseradish peroxidase for 1 h at RT. Plates were washed and incubated with a TMB substrate solution (100 µl/well). After a 10 min-incubation in the dark at RT, the reaction was stopped with the addition of 100 µl of 0.18 M H_2_SO_4_. Optical density of the samples was measured at 450 nm.

### Statistical analysis

To assure that there were differences between naïve and immunized groups, we compared naïve to each immunized group with Student’s t test (for BAL cell data) or Mann-Whitney test (for histological scores). To compare diet and GM effect, the distribution of BAL cell data for all immunized groups was initially tested using Bartlett’s test for equal variances. Because the BAL cell data were not normally distributed, not even after the application of a Box-Cox transformation, we compared them using the Kruskal-Wallis test and means for treatment groups were separated using Dunn’s multiple comparison test. The effects of diet and GM on histological scores were analysed using the Mann-Whitney test. All analyses were done using GraphPad Instat v.5.0 (GraphPad Software Inc.). Values of p were considered significant at <0.05.

## Results

### Allergic airway inflammation

**Figure 2:**
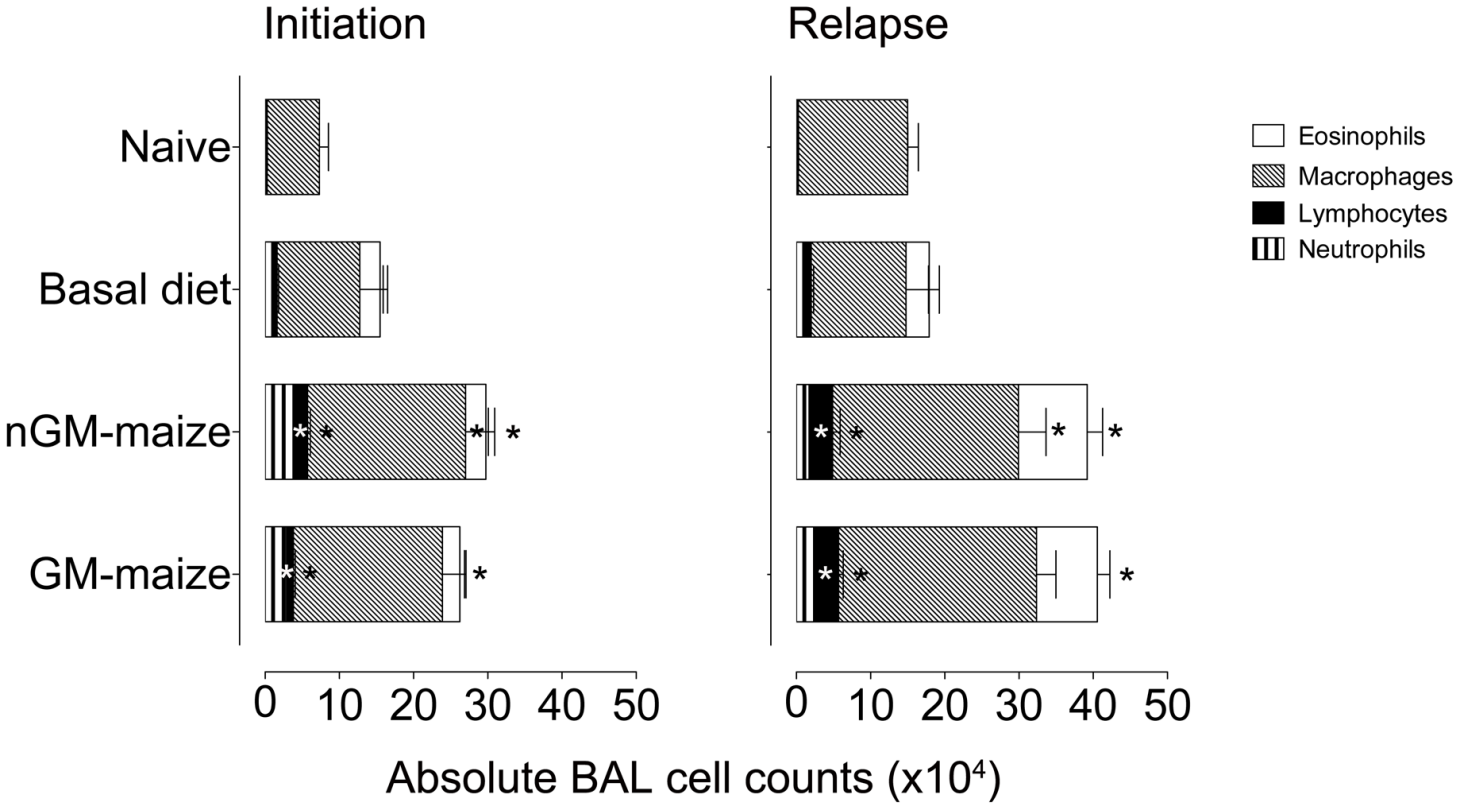
GM-maize consumption does not influence the development of airway allergic inflammation at initiation or relapse of allergic asthma. Three days after the last aerosol challenge, BAL of individual mice was collected and differential cells counts in OVA-induced allergic asthma initiation and during disease relapse were performed. We compared naïve mice vs. mice fed mice basal, GM-maize, and nGM-maize diets for 32–34 days. Representative data from 2 independent experiments are presented as absolute counts of eosinophils, macrophages, neutrophils and lymphocytes and reported as means ± SEM; n = 8. The height of the columns is the total cell count. Kruskal-Wallis test ^*^p<0.05 sample vs. naïve group followed by Dunn’s multiple comparison test.

To evaluate the severity of the inflammatory response in the airways, we enumerated cells in BAL (Fig. 2). Naïve mice had 73333±12561 cells/ml compared with OVA-sensitized and aerosol challenged mice fed the basal diet with 155000±44280 at the induction of allergic disease, whereas mice fed a nGM diet had 297500±48135 and GM diet fed mice had 262500±44631 cells/ml (Fig. 2). This illustrates that immunized and aerosolized groups had approximately 4 times more airway inflammatory cells (p<0.05) than naïve animals and there were no differences between GM- and nGM-fed mice. Upon examination of airway differential counts, we found that mice fed GM (23450±6256 cells/ml) and nGM (27384±11804 cells/ml) diets had significantly higher numbers of eosinophils compared to almost none in naïve mice and that only nGM-fed mice were statistically higher (p<0.05) compared with basal diet-fed mice (27322±10035 cells/ml). The percentage of eosinophils in BAL was 0.1±0.06% for naïve animals with 15.05±3.8% for mice fed the basal diet and 8.29±1.8 and 8.35±1.41% for nGM- and GM-fed groups, respectively. There were also significant increases in macrophages in GM- and nGM-fed groups compared to basal diet-fed mice. We observed increases in neutrophils between nGM- and basal diet-fed mice, but no differences in the numbers of lymphocytes in any of the groups.

At OVA-induced disease relapse, total BAL cell counts were higher than those at the time of disease initiation (Fig. 2). Total BAL cell counts were significantly higher for GM- (405625±47701 cells/ml) and nGM- (409375±64562 cells/ml) fed groups compared to mice fed the basal diet (220000±67447 cells/ml). Moreover, the evaluation of individual cell types demonstrated a significant increase in eosinophils in GM (81927±17088 cells/ml) and nGM (92679±20781 cells/ml) groups compared to basal diet-fed mice (31229±13811 cells/ml). Lymphocytes, neutrophils and macrophages were also significantly increased in mice fed GM and nGM containing diets compared to those fed the basal diet. Taken together, these results illustrate that allergic inflammatory responses were expectedly higher for disease relapse compared with disease initiation. Additionally, these data demonstrate that consuming a high maize containing diet increases OVA-induced allergic airway inflammation and an absence of a *Cry1Ab* effect.

### Allergic lung inflammation

**Figure 3:**
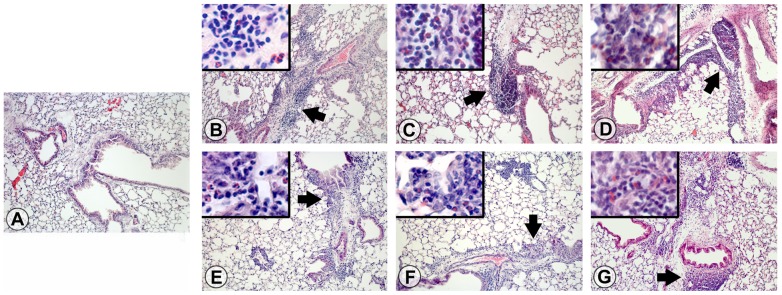
GM-maize consumption does not affect lung inflammation at the initiation or relapse of allergic asthma. Representative photomicrographs of lungs from A) Naïve mice fed a basal diet, and mice at disease initiation fed B) a basal diet, C) nGM-maize, or D) GM-maize, and mice at disease relapse fed, E) a basal diet, F) nGM-maize, or G) GM-maize. H&E-stained lung sections were taken with a 10x objective and are representative data from 2 independent experiments. Arrowheads indicate areas of inflammation and insets are at 40x and illustrate eosinophils within inflammatory infiltrates.

**Figure 4:**
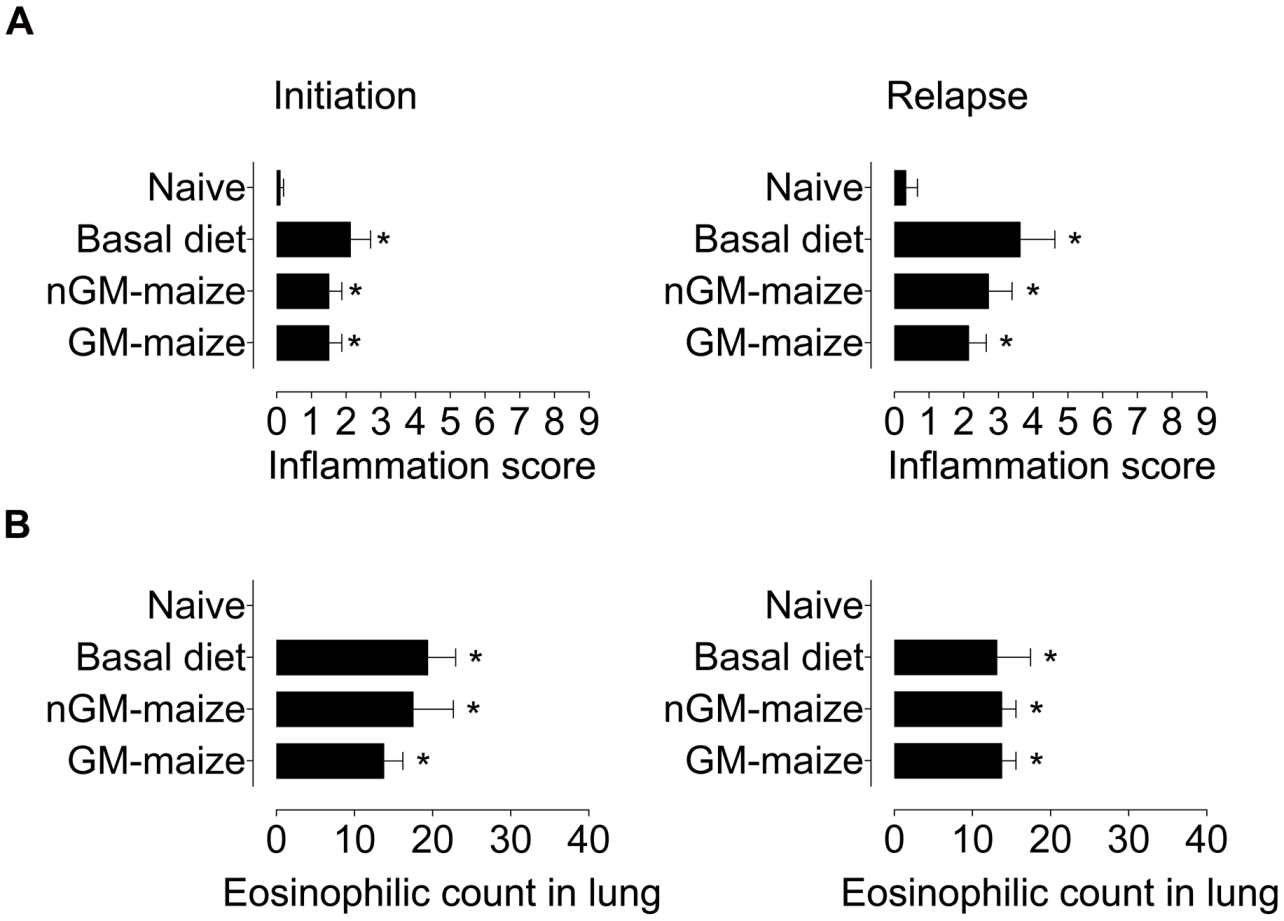
GM-maize consumption does not affect the severity of lung inflammation and the percentage of eosinophils in the lungs at the initiation or relapse of allergic asthma. A) Severity scores graded in H&E-stained lung sections and B) the percentage of eosinophils within infiltrates measured in Luna-stained lung sections from mice at disease initiation and relapse fed diets as shown. These are representative data from 2 independent experiments. Severity scores are reported as means ± SEM; n = 8. Groups are compared using the Kruskal-Wallis test ^*^p<0.05 sample vs. naïve groups.

We evaluated parenchymal inflammation on H&E-stained lung sections from mice fed different diets during the initiation of allergic asthma and before the induction of a disease relapse. Naïve mice had normal parenchyma without any evidence of inflammation (Fig. 3A). All other mice, irrespective of diet, developed intense inflammation throughout the lung parenchyma at disease initiation (Fig. 3B, C, D) and relapse (Fig. 3E, F, G). We observed numerous macrophages and lymphocytes with few neutrophils in infiltrates that were located close to large to medium sized airways in mice administered OVA. Lung sections were scored on the basis of the extent of inflammation and severity and revealed that animals with OVA-induced allergic asthma had significantly (p<0.05) higher scores than the naïve group (Fig. 4A), but no differences were found between OVA-immunized mice fed different diets. In fact, the trend was for higher scores in basal diet-fed mice at disease initiation and relapse. Lung inflammatory infiltrates contained approximately up to 30% eosinophils on Luna-stained sections at disease initiation and relapse (Fig 4B). No significant difference in inflammation was seen in lung parenchyma between groups of OVA-immunized and aerosolized mice fed different diets.

### Mucus secretion in lung

**Figure 5:**
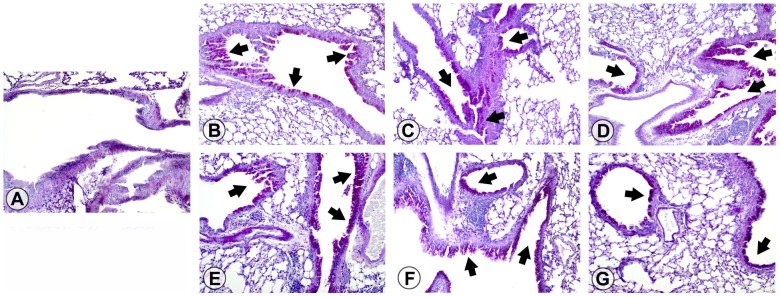
GM-maize consumption does not affect mucus secretion at the initiation or relapse of allergic asthma. Representative photomicrographs of lungs from A) Naïve mice fed a basal diet, and mice at disease initiation fed B) a basal diet, C) nGM-maize, or D) GM-maize, and mice at disease relapse fed, E) a basal diet, F) nGM-maize, or G) GM-maize. Lung PAS-stained sections were observed with a 10x objective. These are representative data from 2 independent experiments. Arrowheads indicate mucus within lung epithelial goblet cells.

**Figure 6:**
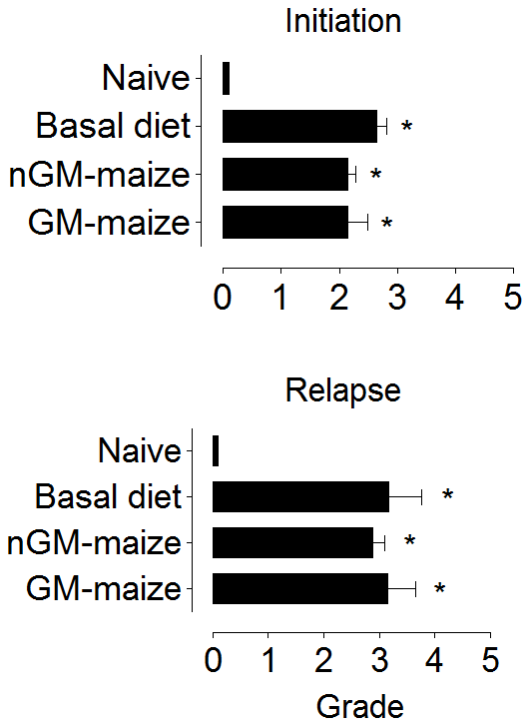
GM-maize consumption does not affect the amount of mucus secreted at initiation or relapse of allergic asthma. Mucus scores graded on PAS-stained lung sections from mice at disease initiation and relapse fed diets as shown. These are representative data from 2 independent experiments. Mucus scores are reported as means ± SEM; n = 8. Groups were compared using the Kruskal-Wallis test ^*^p<0.05 sample vs. naïve groups.

An additional parameter of allergic airways disease is increased production of mucus by lung goblet cells. To determine whether diet influenced mucus secretion, lung sections were stained with PAS. Photomicrographs illustrate few mucus containing goblet cells in naïve mice compared with increased mucus production in all groups immunized and aerosolized with OVA (Fig. 5). Scoring of stained lung sections illustrate that mice fed basal, GM or nGM diets produced similar amounts of mucus (Fig. 6). Taken together, no significant differences in mucus production were found between groups fed different diets at either disease induction or relapse.

### Serum antibody responses

**Figure 7:**
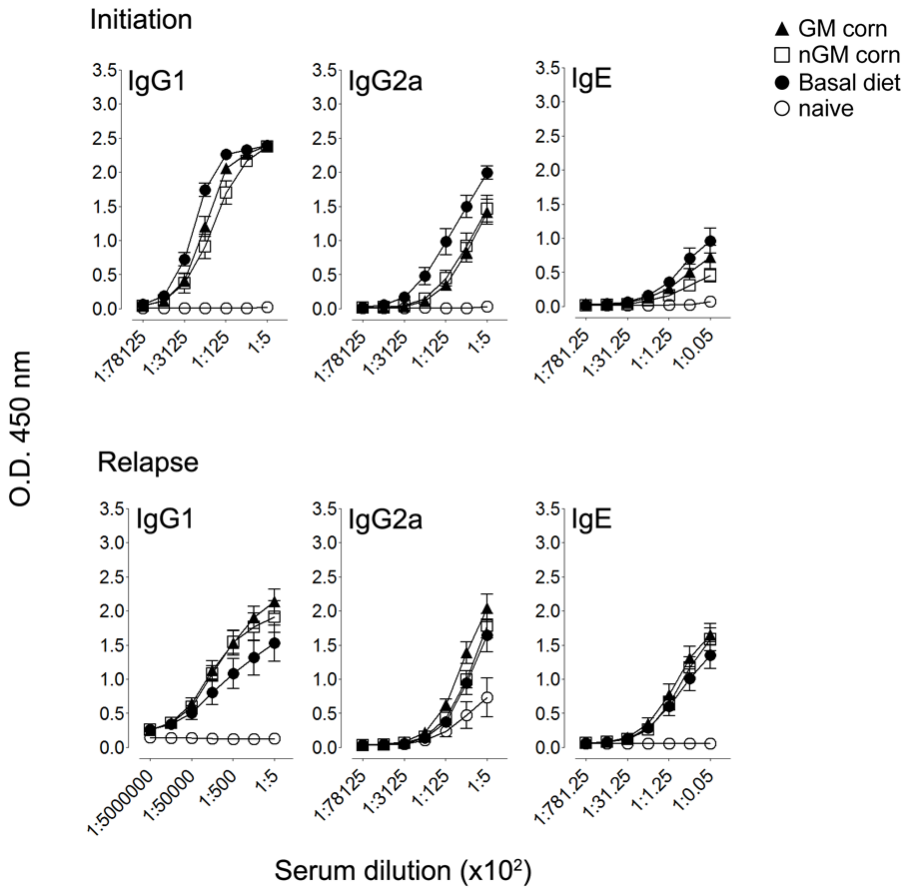
GM-maize consumption does not affect OVA-specific antibody production at the initiation or relapse of allergic asthma. OVA-specific antibody response induced by feeding in mice. Three days after the last aerosol challenge the level of OVA-specific IgG1, IgE and IgG2a antibody response induced by feeding of different diets. Individual serum samples were analyzed for IgG1 and IgE (Th2 phenotype) and IgG2a (Th1 phenotype). Data are expressed as mean O.D. ± SD, n = 8.

An allergic response to OVA immunization and aerosolization generates predominantly OVA-specific IgG1 and IgE antibodies. In unimmunized, naïve mice, no antibody titres were observed (Fig. 7). However, all immunized mice irrespective of diet had high titres of OVA-specific IgG1 and lower titres of IgE at disease initiation and relapse, but there were no significant differences in antibody titres. This protocol is skewed towards allergic Th2-type responses, but the mice also developed Th1-type OVA-specific IgG2a antibodies, but no differences were seen between groups. These data illustrate an absence of an adjuvant effect of GM-maize consumption on antibody production. Because the maize-based diets contained milk proteins not included in the basal diet, it is possible that the novel milk proteins that are co-ingested would generate an antibody response that might be augmented with an adjuvant. To test this possibility, we measured serum IgG1antibody responses against the BSA component in cow’s milk and whole milk. We found no differences in serum IgG1 titres between animals fed basal, GM and nGM diets (Fig. S1).

## Discussion

Some investigators suggest that *Bt*-maize may have an adjuvant effect that requires testing [17]–[20], [26]–[30]. In 2009, EFSA reported that consumption of *Bt*-maize by humans and animals is safe [14]. The Codex Alimentarius Commission recommendations indicate that the weight of evidence approach during pre-market assessment should suffice for the determination of adjuvanticity of a GM crop [31]–[33]. Here, we show the absence of an adjuvant effect of *Bt*-maize consumption in mice at initiation and relapse of OVA-induced allergic asthma. Airway and lung inflammation, mucus hypersecretion, and antibody titres were not influenced upon consumption of *Bt*-maize. Thus, in our studies, *Bt*-maize consumption did not augment the elicitation of allergic responses to unrelated allergens or exacerbate allergic responses in mice with pre-existing allergy. Taken together these data demonstrate that *Bt*-maize does not act as an immunological adjuvant.

We hypothesized that for testing GM foods for adjuvanticity, it is important to evaluate the GM upon consumption of the food. Using this approach in the current study differed from previous studies in that purified proteins were tested instead of the whole food. For example, there is one report in which allergic sensitization and elicitation to peanut in mice revealed *Cry1Ab* adjuvanticity on the elicitation of an allergic response to another protein when *Cry1Ab* was gavaged to mice at high concentrations [20]. Most experiments that reportedly showed adjuvanticity of Cry proteins focused on the *Cry1Ac* protoxin and not the mature, activated form of *Cry1Ab*. In one study, *Cry1Ac* was investigated for *in vivo* activation of a variety of macrophages after intraperitoneal or intranasal administration in BALB/c mice. The protoxin upregulated costimulatory molecules on macrophages and enhanced the production of the TNF-α, IL-6, and MCP-1 [26]. In another study, *Cry1Ac* delivered intranasally generated an antibody response, stimulated nasal-associated lymphoid tissue lymphocytes to produce Th2 cytokines, and induced a proportion of T cells to spontaneously produce IL-4, IL-5, and IL-10 [27]. Indeed several studies have shown that *Cry1Ac* has adjuvant effects using non-physiological approaches [28]–[30] and it is not clear from these reports whether *Cry1Ac* effects also apply to *Cry1Ab*.

In gavage experiments with purified protein, the precise amount of *Cry1Ab* is known, however, when the mice are provided with *ad libitum* access to a diet of *Bt*-maize, the level of *Cry1Ab* delivered is more difficult to evaluate. Mice eat approximately 4.4 g of food per day and in our study, they consumed sample diets for 32 or 34 consecutive days. The mice were provided a diet including 33% *Bt*-maize containing approximately 0.0013% *Cry1Ab* [25]. Thus, in total, each animal consumed either 600 or 641 µg of *Cry1Ab* during the experiment. It is possible that protein denaturation of *Cry1Ab* during food processing might reduce the amount of *Cry1Ab* in the food, but this is very unlikely because steam pelleting at 70°C is a gentle process. Nevertheless, the amount of *Cry1Ab* ingested from a steady diet of *Bt*-maize including potential denaturation of the protein is likely to be higher than previous studies using gavage in which 100 µg of *Cry1Ab* was provided on days 0 and 15 in one study [25] and in another study, mice were gavaged with a total of 50 µg of *Cry1Ab* on 5 different days [20]. Compared to gavage treatment with purified protein, mice in our experiments consumed similar or even higher amounts of *Cry1Ab* without adjuvant effects.

Our study shows that not only does *Bt*-maize consumption not influence the induction of an allergic response to an unrelated protein like OVA, there is no effect on the exacerbation of an allergic response in an animal with pre-existing allergy. These results show that consumption does not influence the response to allergen provided systemically and to the respiratory tract. Moreover, there was a lack of an adjuvant effect in mice having ingested cow’s milk proteins contained in the maize diets compared with the basal diet. These results differ from data generated using an experimental mouse model in which a response is induced against milk proteins upon the co-administration of cow’s milk and cholera toxin [34].

Previously, the potential for a GM food-induced adjuvant effect was reported with a GM food unrelated to *Bt* [21]. Prescott et al. found that GM alpha-amylase inhibitor (AAI) peas had an adjuvant effect on non-cross-reactive OVA responses. However, in a more comprehensive study, we demonstrated the absence of an adjuvant effect also using AAI peas in the same experimental model reported herein [22]. Additionally, we found that a cross-reactive allergic response against pea lectin and AAI developed upon AAI and nGM pea consumption. However, despite this immune response, there was no adjuvant effect of the AAI peas. In our current study, we observed significant increases in inflammation in the airways during the consumption of maize–based diets (both GM and nGM) compared with the conventional, basal diet. This may suggest that there are additional ingredients between the maize-containing and basal diets such as lectins, which may have adjuvanticity. The observation that maize and basal diet-fed mice exhibited similar parenchymal lung inflammation may relate to differences in the time to peak inflammation for lung vs. airway inflammatory responses.

In summary, our current study indicates that consumption of a *Bt*-maize containing diet did not influence allergic responses to the experimental, unrelated OVA-induced disease initiation and relapse of allergic asthma. This study differs from previous studies in that the mice were provided *Bt*- and nGM maize included in their diets, which is physiological and more relevant than administering purified Cry proteins via alternative routes. Taken together, our data show that there is no adjuvant effect on an allergic response to a non-crossreactive protein upon *Bt*-maize (Cry1Ab) consumption in a mouse model.

## Supporting Information

Figure S1 The effect of co-ingestion of GM– and nGM– maize and milk proteins on anti-milk– and BSA– specific IgG1 titres in serum. Milk– and BSA– specific antibody titres in mice fed basal, GM and nGM diets. Following 32 (initiation) and 34 (relapse) days of mice fed basal, GM and nGM diets, sera were tested for IgG1. Experimental groups were compared with sera collected from mice immunized i.p. with either milk or BSA. Data are expressed as mean O.D. ± SEM, n = 8.
(TIF)

## References

[1] James C (2012) Global Status of Commercialized Biotech/GM Crops: 2012. ISAAA Brief No.44.

[2] Crickmore N, Zeigler DR, Feitelson J, Schnepf E, Van Rie J, et al (1998) Revision of the nomenclature for the Bacillus thuringiensis pesticidal crystal proteins. Microbiology and molecular biology reviews : MMBR 62: 807–813. 9729610 10.1128/mmbr.62.3.807-813.1998 PMC98935

[3] Broderick NA, Robinson CJ, McMahon MD, Holt J, Handelsman J, et al (2009) Contributions of gut bacteria to Bacillus thuringiensis-induced mortality vary across a range of Lepidoptera. BMC Biol 7: 11. 19261175 10.1186/1741-7007-7-11 PMC2653032

[4] Crickmore N (2005) Using worms to better understand how Bacillus thuringiensis kills insects. Trends Microbiol 13: 347–350. 15967665 10.1016/j.tim.2005.06.002

[5] Flachowsky G, Aulrich K, Bohme H, Halle I (2007) Studies on feeds from genetically modified plants (GMP) - Contributions to nutritional and safety assessment. Animal feed science and technology 133: 2–30.

[6] Sanden M, Krogdahl A, Bakke-McKellep AM, Buddington RK, Hemre GI (2006) Growth performance and organ development in Atlantic salmon, *Salmo salar* L. parr fed genetically modified (GM) soybean and maize. Aquaculture Nutrition 12: 1–14.

[7] Trabalza-Marinucci M, Brandi G, Rondini C, Avellini L, Giammarini C, et al (2008) A three-year longitudinal study on the effects of a diet containing genetically modified Bt176 maize on the health status and performance of sheep. Livestock Science 113: 178–190.

[8] Custodio MG, Powers WJ, Huff-Longergan E, Faust MA, Stein J (2006) Growth, pork quality, and excretion characteristics of pigs fed BT corn or non-transgenic corn. Canadian Journal of Animal Science 86: 461–469.

[9] Reuter T, Aulrich K, Berk A (2002) Investigations on genetically modified maize (Bt-maize) in pig nutrition: fattening performance and slaughtering results. Arch Tierernahr 56: 319–326. 12556043 10.1080/00039420215628

[10] Sanden M, Berntssen MH, Krogdahl A, Hemre GI, Bakke-McKellep AM (2005) An examination of the intestinal tract of Atlantic salmon, Salmo salar L., parr fed different varieties of soy and maize. J Fish Dis 28: 317–330. 15960655 10.1111/j.1365-2761.2005.00618.x

[11] Shimada N, Murata H, Mikami O, Yoshioka M, Guruge KS, et al (2006) Effects of feeding calves genetically modified corn bt11: a clinico-biochemical study. J Vet Med Sci 68: 1113–1115. 17085894 10.1292/jvms.68.1113

[12] Kilic A, Akay MT (2008) A three generation study with genetically modified Bt corn in rats: Biochemical and histopathological investigation. Food and chemical toxicology : an international journal published for the British Industrial Biological Research Association 46: 1164–1170. 18191319 10.1016/j.fct.2007.11.016

[13] Finamore A, Roselli M, Britti S, Monastra G, Ambra R, et al (2008) Intestinal and peripheral immune response to MON810 maize ingestion in weaning and old mice. J Agric Food Chem 56: 11533–11539. 19007233 10.1021/jf802059w

[14] EFSA (2009) Scientific opinion of the panel on genetically modified organisms. EFSA Journal 1149.

[15] Batista R, Nunes B, Carmo M, Cardoso C, Jose HS, et al (2005) Lack of detectable allergenicity of transgenic maize and soya samples. The Journal of allergy and clinical immunology 116: 403–410. 16083797 10.1016/j.jaci.2005.04.014

[16] Kroghsbo S, Madsen C, Poulsen M, Schroder M, Kvist PH, et al (2008) Immunotoxicological studies of genetically modified rice expressing PHA-E lectin or Bt toxin in Wistar rats. Toxicology 245: 24–34. 18215453 10.1016/j.tox.2007.12.005

[17] Vazquez-Padron RI, Moreno-Fierros L, Neri-Bazan L, Martinez-Gil AF, de-la-Riva GA, et al (2000) Characterization of the mucosal and systemic immune response induced by Cry1Ac protein from Bacillus thuringiensis HD 73 in mice. Brazilian journal of medical and biological research = Revista brasileira de pesquisas medicas e biologicas/Sociedade Brasileira de Biofisica [et al] 33: 147–155. 10.1590/s0100-879x2000000200002 10657055

[18] Moreno-Fierros L, Ruiz-Medina EJ, Esquivel R, Lopez-Revilla R, Pina-Cruz S (2003) Intranasal Cry1Ac protoxin is an effective mucosal and systemic carrier and adjuvant of Streptococcus pneumoniae polysaccharides in mice. Scandinavian journal of immunology 57: 45–55. 12542797 10.1046/j.1365-3083.2003.01190.x

[19] Rojas-Hernandez S, Rodriguez-Monroy MA, Lopez-Revilla R, Resendiz-Albor AA, Moreno-Fierros L (2004) Intranasal coadministration of the Cry1Ac protoxin with amoebal lysates increases protection against Naegleria fowleri meningoencephalitis. Infection and immunity 72: 4368–4375. 15271892 10.1128/IAI.72.8.4368-4375.2004 PMC470623

[20] Guimaraes VD, Drumare M-F, Ah-Leung S, Lereclus D, Bernard H, et al (2008) Comparative study of the adjuvanticity of Bacillus thuringiensis Cry1Ab protein and cholera toxin on allergic sensitisation and elicitation to peanut. Food and Agricultural Immunology 19: 325–337.

[21] Prescott VE, Campbell PM, Moore A, Mattes J, Rothenberg ME, et al (2005) Transgenic expression of bean alpha-amylase inhibitor in peas results in altered structure and immunogenicity. Journal of agricultural and food chemistry 53: 9023–9030. 16277398 10.1021/jf050594v

[22] Lee RY, Reiner D, Dekan G, Moore AE, Higgins TJ, et al (2013) Genetically modified alpha-amylase inhibitor peas are not specifically allergenic in mice. PloS one 8: e52972. 23326368 10.1371/journal.pone.0052972 PMC3541390

[23] Mojtabavi N, Dekan G, Stingl G, Epstein MM (2002) Long-lived Th2 memory in experimental allergic asthma. Journal of immunology 169: 4788–4796. 10.4049/jimmunol.169.9.4788 12391188

[24] Walsh MC, Buzoianu SG, Gardiner GE, Rea MC, Ross RP, et al (2012) Effects of short-term feeding of Bt MON810 maize on growth performance, organ morphology and function in pigs. The British journal of nutrition 107: 364–371. 21733303 10.1017/S0007114511003011

[25] Adel-Patient K, Guimaraes VD, Paris A, Drumare MF, Ah-Leung S, et al (2011) Immunological and metabolomic impacts of administration of Cry1Ab protein and MON 810 maize in mouse. PloS one 6: e16346. 21298004 10.1371/journal.pone.0016346 PMC3029317

[26] Moreno-Fierros L, Garcia-Hernandez AL, Ilhuicatzi-Alvarado D, Rivera-Santiago L, Torres-Martinez M, et al (2013) Cry1Ac protoxin from Bacillus thuringiensis promotes macrophage activation by upregulating CD80 and CD86 and by inducing IL-6, MCP-1 and TNF-alpha cytokines. International immunopharmacology 17: 1051–1066. 24157331 10.1016/j.intimp.2013.10.005

[27] Rodriguez-Monroy MA, Moreno-Fierros L (2010) Striking activation of NALT and nasal passages lymphocytes induced by intranasal immunization with Cry1Ac protoxin. Scandinavian journal of immunology 71: 159–168. 20415781 10.1111/j.1365-3083.2009.02358.x

[28] Legorreta-Herrera M, Meza RO, Moreno-Fierros L (2010) Pretreatment with Cry1Ac protoxin modulates the immune response, and increases the survival of Plasmodium-infected CBA/Ca mice. Journal of biomedicine & biotechnology 2010: 198921. 20300584 10.1155/2010/198921 PMC2837902

[29] Verdin-Teran SL, Vilches-Flores A, Moreno-Fierros L (2009) Immunization with Cry1Ac from Bacillus thuringiensis increases intestinal IgG response and induces the expression of FcRn in the intestinal epithelium of adult mice. Scandinavian journal of immunology 70: 596–607. 19906202 10.1111/j.1365-3083.2009.02332.x PMC7169514

[30] Carrasco-Yepez M, Rojas-Hernandez S, Rodriguez-Monroy MA, Terrazas LI, Moreno-Fierros L (2010) Protection against Naegleria fowleri infection in mice immunized with Cry1Ac plus amoebic lysates is dependent on the STAT6 Th2 response. Parasite immunology 32: 664–670. 20691018 10.1111/j.1365-3024.2010.01222.x

[31] Programme JFWFS (2003) Twenty-Fifth Session, Rome, Italy 30 June-5 July, 2003. Appendix III, Guideline for the conduct of food safety assessment of foods derived from recombinant-DNA plants and Appendix IV, Annex on the assessment of possible allergenicity. Codex Alimentarius Commission 5: 47–60.

[32] EFSA (2006) EFSA Guidance document of the Scientific Panel on Genetically Modified Organisms for the risk assessment of genetically modified microorganisms and their derived products intended for food and feed use. EFSA Journal 374: 1–115.

[33] Organisms EPoGM (2011) Guidance on the risk assessment of genetically modified microorganisms and their products intended for food and feed use. EFSA Journal 9: 2193.

[34] Adel-Patient K, Bernard H, Ah-Leung S, Creminon C, Wal JM (2005) Peanut- and cow’s milk-specific IgE, Th2 cells and local anaphylactic reaction are induced in Balb/c mice orally sensitized with cholera toxin. Allergy 60: 658–664. 15813812 10.1111/j.1398-9995.2005.00767.x

