# Supplementary figures and images for: No Adjuvant Effect of Bacillus thuringiensis-Maize on Allergic Responses in Mice
Source: PLoS One. 2014 Aug 1;9(8):e103979. doi: 10.1371/journal.pone.0103979 (PMC4118972; doi:10.1371/journal.pone.0103979)

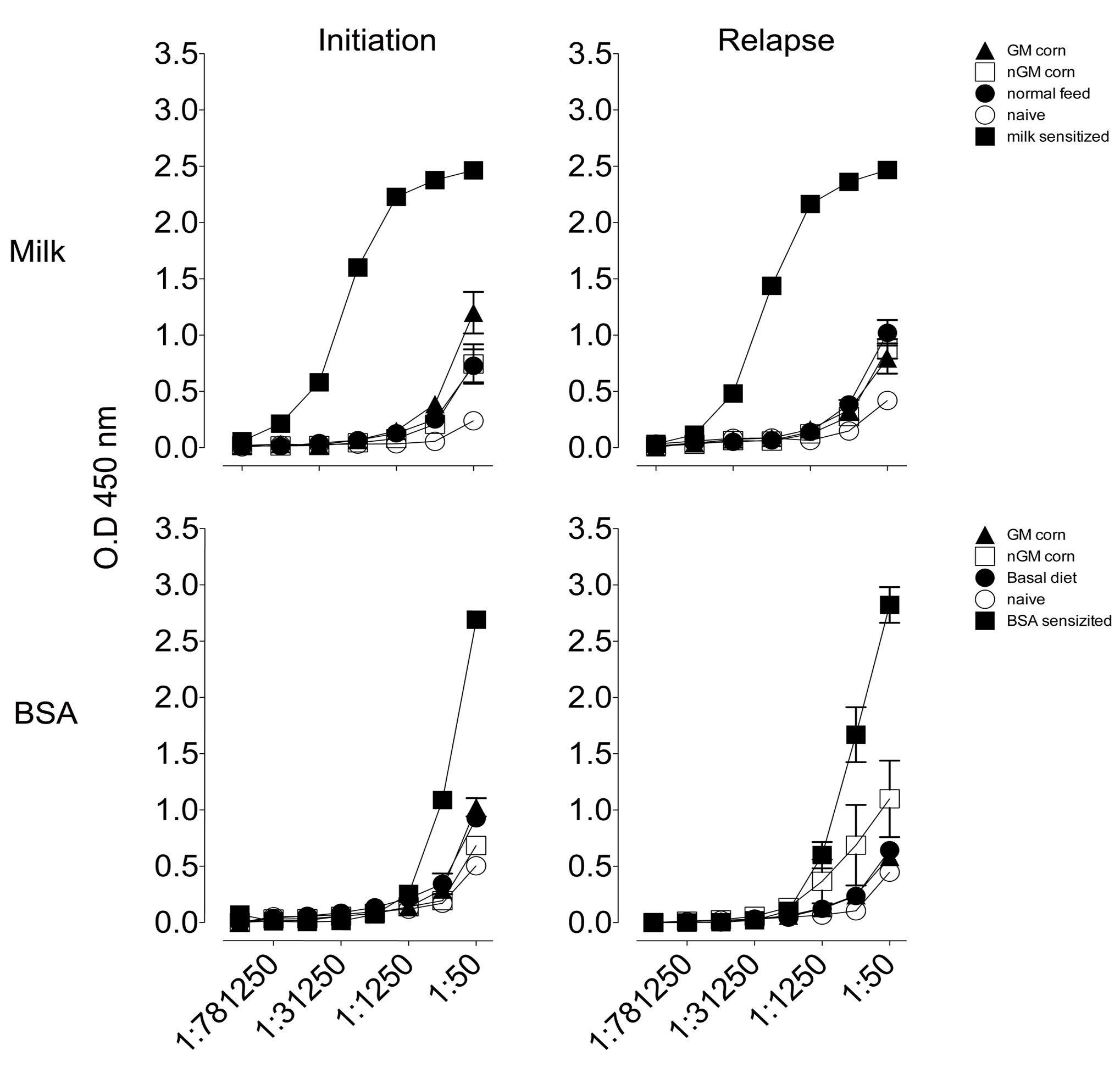

Supplement: Figure S1 — The effect of co-ingestion of GM– and nGM– maize and milk proteins on anti-milk– and BSA– specific IgG1 titres in serum. Milk– and BSA– specific antibody titres in mice fed basal, GM and nGM diets. Following 32 (initiation) and 34 (relapse) days of mice fed basal, GM and nGM diets, sera were tested for IgG1. Experimental groups were compared with sera collected from mice immunized i.p. with either milk or BSA. Data are expressed as mean O.D. ± SEM, n = 8. (TIF) [file pone.0103979.s001.tif]
